# The expansion of the metazoan microRNA repertoire

**DOI:** 10.1186/1471-2164-7-25

**Published:** 2006-02-15

**Authors:** Jana Hertel, Manuela Lindemeyer, Kristin Missal, Claudia Fried, Andrea Tanzer, Christoph Flamm, Ivo L Hofacker, Peter F Stadler, The Students of Bioinformatics Computer Labs 2004 and 2005

**Affiliations:** 1 Bioinformatics Group, Department of Computer Science, University of Leipzig, Härtelstrasse 16-18, D-04107 Leipzig, Germany; 2 Institute for Theoretical Chemistry, University of Vienna, Währingerstrasse 17, A-1090 Wien, Austria; 3 The Santa Fe Institute, 1399 Hyde Park Rd., Santa Fe NM 87501

## Abstract

**Background:**

MicroRNAs have been identified as crucial regulators in both animals and plants. Here we report on a comprehensive comparative study of all known miRNA families in animals. We expand the MicroRNA Registry 6.0 by more than 1000 new homologs of miRNA precursors whose expression has been verified in at least one species. Using this uniform data basis we analyze their evolutionary history in terms of individual gene phylogenies and in terms of preservation of genomic nearness across species. This allows us to reliably identify microRNA clusters that are derived from a common transcript.

**Results:**

We identify three episodes of microRNA innovation that correspond to major developmental innovations: A class of about 20 miRNAs is common to protostomes and deuterostomes and might be related to the advent of bilaterians. A second large wave of innovations maps to the branch leading to the vertebrates. The third significant outburst of miRNA innovation coincides with placental (eutherian) mammals. In addition, we observe the expected expansion of the microRNA inventory due to genome duplications in early vertebrates and in an ancestral teleost. The non-local duplications in the vertebrate ancestor are predated by local (tandem) duplications leading to the formation of about a dozen ancient microRNA clusters.

**Conclusion:**

Our results suggest that microRNA innovation is an ongoing process. Major expansions of the metazoan miRNA repertoire coincide with the advent of bilaterians, vertebrates, and (placental) mammals.

## Background

MicroRNAs (miRNAs) are small non-coding RNAs that can be found in both multi-cellular animals and plants. In both kingdoms they act as negative regulators of translation. They are transcribed as longer primary transcripts from which approximately 70nt precursors (pre-miRNAs) with a characteristic stem-loop structure are extracted; after export to the cytoplasm, the mature miRNAs, approximately 22nt in length, are cut out from one side of the precursor stem structure. For reviews on the discovery and function of miRNAs we refer to the literature, see e.g. [1,2].

Despite the rapid growth of our knowledge on microRNA regulation, little is known about the evolution and phylogenetic distribution of the hundreds of animal microRNA families. The exceptions are a few well-studied examples, including *let-7 *[3-5], the three non-homologous miRNA families comprising the **mir-17 **cluster [6,7], two *Hox*-cluster associated genes *mir-10 *and *mir-196 *[8,9], and the exceptional imprinted **mir-134 **cluster of microRNAs located at human locus 14q32 [10-12]. These few case studies, which were selected because of special properties of the miRNAs in question, of course cannot provide a comprehensive, or even representative, picture of microRNA evolution in animals.

Two very recent papers discuss in detail the phylogenetic distribution of plant microRNAs using expression profiling [13] and EST data [14], respectively. Both studies demonstrate that "several individual miRNA regulatory circuits have ancient origins and have remained intact throughout the evolution and diversification of plants." With only a limited number of miRNA families to investigate (17 in [14] and 23 in [13]) the situation is much more favorable than in animals, where the MicroRNA Registry 6.0 (MR 6.0) [15,16] lists more than 1200 microRNAs which fall into more than 300 families defined by their "mir-number" [17]. A recent comprehensive study of microRNA gene expression in zebrafish [18], for example, lists 142 miRNA loci in the genome of *Danio rerio *that are homologous to more than 100 different mammalian microRNAs, belonging to almost 100 different families.

In this contribution we report on a comprehensive study of the phylogenetic distribution and evolutionary histories of the currently known miRNAs (as defined by the content of version 6.0 of the MicroRNA Registry) and their homologs.

## Results

### Novel microRNA genes

While microRNAs have been studied in much detail in mammals, insects, and nematodes, much less is known in other lineages. Information on chicken, frog, and actinopterygian microRNAs are almost exclusively based on sequence homology. In this study we have attempted to obtain this information systematically and as exhaustively as possible. To this end, we include only those predicted microRNA candidates which can be identified as homologs of a MR 6.0 entry. Note that our statistics ignores all microRNAs that are not contained in MR 6.0, most notably, many of those reported in recent studies of primates [19,20] and zebrafish [18,21]. While a recent survey for ncRNAs has provided evidence for a significant number of microRNAs in *Ciona intestinalis *[22], most of them are not included here because their homology with known vertebrate microRNAs cannot be established unambiguously.

Table 1 summarizes the microRNA precursor sequences that form the basis for this study, a detailed list is provided in additional file: 1; insect-specific microRNAs are summarized in additional file: 2 (see supplemental material).

Our knowledge of microRNAs in basal deuterostomes is sketchy at best, despite the fact that four genomes are available at various stages of completion. In this survey we detect a number of microRNAs in basal deuterostomes: 40 sequences in only 6 families (*mir-1*, *mir-9*, *mir-31*, *mir-124*, *mir-125*, *mir-184*) were found in the genome of the sea urchin *Strongylocentrotus purpuratus*. Most of the 40 sequences will probably turn out to be identical in more advanced assemblies of the genome. A handful of families were detected in urochordates. In [22], 41 putative microRNAs are predicted in *Ciona intestinalis*, of which only 4 are recognizable orthologs of known vertebrate microRNAs. It is not clear whether the other candidates are lineage-specific innovations, or whether they are too diverged to recognize their homology with known microRNA families.

Similarly, we find only three convincing microRNA candidates in the trematode *Schistosoma mansoni*: *mir-1*, *mir-9*, and *mir-124*. In contrast, no plausible orthologs were detected outside the metazoa e.g. in *Schizosaccharomyzes pombe *or *Encephalitozoon cuniculi*.

### Phylogenetic distribution of microRNA families

The tables in additional file: 1 as well as in the summary of microRNA precursor sequences, both part of the extensive electronic supplement  summarize the sequences that were found through the combined blast and erpin searches described above. Since large-scale experimental surveys that were not based on *a priori *homology information have been performed only for 4 species (*Homo sapiens*, *Mus musculus*, *Drosophila melanogaster*, *Caenorhabditis elegans*) we can only analyze the innovation of microRNAs along the branches of the phylogenetic tree leading to those four species.

To this end, we map each miRNA to the branch that leads to the last common ancestor of all homologs that we could identify in our survey. Note that this does not imply that all children of this ancestral node carry a known homolog: miRNAs may have been lost in a particular lineage or they may have diverged too far to be recognizable by homology-based searches. We suspect that the small number of identified miRNAs in basal deuterostome (both *Strongylocentrotus purpuratus *and the urochordates) and in *Schistosoma mansoni *is predominantly due to sequence divergence rather than true gene loss.

To our surprise, we find that miRNA innovation is an ongoing process, exemplified already by the small number of rodent or primate-specific sequences contained in MR 6.0. Recent studies by Berezikov *et al*. [19] and Bentwich *et al*. [20] demonstrate that evolutionary young miRNAs are a common phenomenon. Many of these are members of large miRNA clusters. Note that our data set contains at least one representative of many of these clusters, suggesting that expansion of existing clusters is a major mode of miRNA evolution. On the other hand, we can clearly identify two edges in the phylogenetic tree along which innovation is concentrated: the edge leading to the ancestral gnathostome, and the edge leading to the ancestral eutherian.

In addition to the introduction of a large number of novel miRNA sequences, we find a large number of paralogous miRNA sequences throughout the metazoa. Two classes of duplication events are easily distinguishable:

• Local (tandem) duplications result in paralogous sequences that are (typically) located on the same transcript. These gene copies retain their physical linkage over long evolutionary timescales.

• Non-local duplications result in paralogous genes (or gene clusters) on (usually) different chromosomes. In some cases, copies on the same chromosome separated by large distances are observed, but in these cases the physical linkage is not preserved across larger evolutionary times.

Non-local duplications almost exclusively can be allocated to only two points in the metazoan phylogeny: in the stem of the teleost branch and in the edge separating the gnathostome ancestor from the urochordates. This is consistent with the large-scale, probably genome-wide, duplications postulated by the 2R/3R model [23-25].

As expected, we find no case of a microRNA family with more than 4 different genomic loci in tetrapods or more than 8 genomic loci in teleosts, with the sole exception of the *let-7 *family. In this case, which was studied in detail in [5], at least one non-local duplication event predates vertebrate-specific genome duplications.

Indeed, we find that about 50% of the isolated microRNAs or microRNA clusters that predate the last common ancestor of tetrapods and teleosts appear in at least two separate genomic loci. Similarly, about 50% of these "old" microRNAs show clear evidence for an additional duplication of at least one copy in the teleosts lineage.

### MicroRNA clusters

A substantial fraction of microRNAs are located on polycistronic transcripts [26-29]. Tab. 2 lists the vertebrate microRNA clusters. MicroRNA clustering is also a common phenomenon in invertebrates: (see summary table in additional file: 2, supplemental material). The evolutionary history of four microRNA clusters has already been described in detail in the literature:

Probably the best-understood microRNA, at least in terms of its phylogenetic distribution is *let-7*, which was discovered in *C. elegans *as a timing regulator in development [30]. The *let-7 *microRNA is present in diverse animal phyla including chordates, echinoderms, mollusks, annelids, arthropods, nematodes, chaetognaths, nemerteans, and platyhelminths, but it is absent in basal metazoa including cnidarians, poriferans, ctenophora, and acoel flatworms [3,4]. In vertebrates a plethora of *let-7 *paralogs are known. Paralogs of the two miRNAs *mir-100 *and *mir-125 *are transcribed together with some of the *let-7 *paralogs in both vertebrates and insects. For a detailed reconstruction of the *let-7 *gene phylogeny we refer to [5].

The **mir-17 **cluster consists of up to 6 members belonging to three non-homologous microRNA families: *mir-17*, *mir-19*, and *mir-92*. While *mir-92 *can easily be traced back to the common ancestor of protostomes and deuterostomes, the other two families appear to be younger [6].

The **mir-134 **cluster is a unique system of microRNAs located at the imprinted human locus 14q32 [10-12,31] and the orthologous mouse *Dlkl-Gtl2 *domain [32]. It is restricted to eutherian mammals and consists of 6 known groups of microRNAs, which, however, according to our analysis share a common origin, see Fig. 7 below. The most prolific subgroup consists of *mir-154 *and its paralogs, which appear to be rapidly radiating. Local sub-clusters of this unique system are studied in detail in [33]. These authors also report additional cluster members that are not contained in the MR 6.0.

The **mir-290 **cluster consists of murine microRNAs *mir-290 *to *mir-295 *and their human homologs *mir-371 *to *mir-373*. It is conserved in eutherian mammals and is rapidly evolving both in gene content and sequence [20,34].

**Figure 3:**
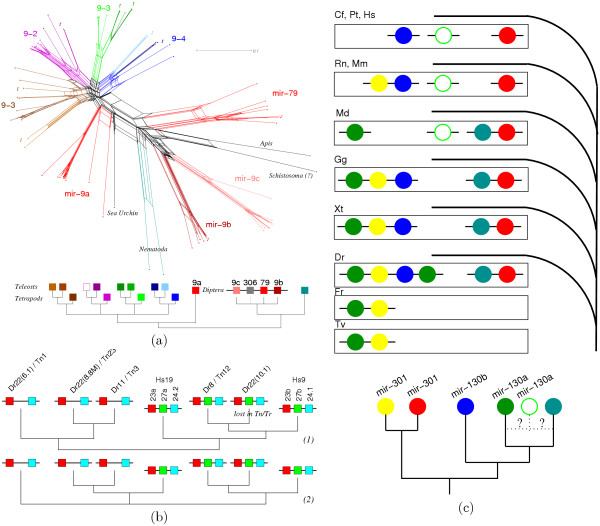
Examples of microRNA gene duplication histories. (a) Gene tree and most plausible reconstructed history of the **mir9 **cluster. The fourth member of the cluster, *mir-306*, evolves rapidly in flies. Its homology with *mir-9*/*mir-79 *is likely but this hairpin might also have evolved *de novo*. (b) The two most plausible reconstructions for the history of the **mir-23 **cluster. Scenario (1) postulates four paralogs in the ancestral vertebrate, where, presumably after the first duplication, one lineage either lost or gained *mir-27 *in the middle position of the cluster. Subsequently, in this scenario one copy of the three-membered cluster was lost in actinopterygians, while the two-membered clusters were lost in tetrapoda. Scenario (2) postulates three paralogs in the ancestral vertebrate and the independent loss of the *mir-27 *in two distinct clusters in the teleosts. (c) Duplication history of the **mir-130 **cluster reconstructed from genomic position information and the gene tree.

Other miRNA clusters have not been analyzed in detail to our knowledge. Our own finding are summarized below, see also Fig. 3. Gene phylogenies of all microRNA families are provided in the supplemental material.

The **mir-1 **cluster is ancient, consisting of *mir-1 *and *mir-133*; (except in nematodes where *mir-133 *seems to be absent). In vertebrates, there are three copies on different chromosomes.

The *mir-9 *family is also ancient. In diptera, we have both an isolated *mir-9 *paralog (most closely related to its vertebrate homologs) and a cluster of four microRNAs consisting of *mir-9c*, *mir-306*, *mir-79*, and *mir-9b*, see Fig. 3a. This cluster, which presumably arose by means of tandem duplications, is specific to diptera. One of the four members of this **mir-9 **cluster, *mir-306*, is so diverged that its homology with *mir-9/mir-79 *is not unambiguous.

The **mir-15 **cluster arose from an old tandem duplication. It occurs in 3 copies in tetrapoda, were one locus has only a single copy of the microRNA.

**Figure 4:**
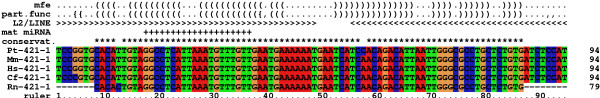
Clustalw multiple sequence alignment of *mir-421 *homologs on the mammalian X chromosome. Additional features (top down): mfe: minimum free energy structure calculated using RNAfold -d2 -noLP, part. func: partition function fold, L2/LINE: direction and position of L2 elements relative to *mir-421*, mat miRNA: position of mature miRNA, conservat.: conserved positions in sequence alignment.

In some cases, even the combination of sequence information and physical linkage is insufficient to completely resolve the history of a microRNA cluster. As an example, consider the **mir-23 **cluster, consisting of *mir-23*, *mir-24*, and *mir-27*, which appear to have unrelated sequences. While tetrapoda have two clusters consisting of all three miRNAs, teleost fishes have either four (pufferfishes) or five (zebrafish) copies, usually on different chromosomes or at least separated several million bases from each other. Fig. 4 gives the two most plausible scenarios, both of which are based on the assumption of the 2R/3R model that leads us to expect up to four paralogs in the ancestral vertebrate and a duplication of this ancestral state in the teleosts.

The **mir-141 **cluster consists of the paralogous microRNAs *mir-141 *and *mir-200*. The ancient tandem duplication that created this cluster predates the origin of the chordates (but there do not seem to be homologous arthropod or nematode sequences). In vertebrates there are two copies of the clusters.

The **mir-302 **cluster consists of four tandem copies of *mir-302 *and a single copy of *mir-367 *in amniotes. Homologs in more distant groups, including frog and teleosts, could not be identified.

A small number of microRNA clusters arose only recently, i.e., after the last common ancestor of eutherian mammals. For example, *mir-298 *arose next to *mir-296 *in the rodent lineage. *mir-105*, which is located on the X-chromosome, exists in three copies in *Canis *and in two copies in *Homo*, while other mammals have only a single copy.

Conversely, a few ancient microRNA families have be remodeled considerably in mammals. The **mir-130 **cluster, Fig. 3c, may serve as an example. This family arose by tandem duplications very early in vertebrates. An additional copy appears early in the mammalian lineage followed by different lineage specific deletions.

### MicroRNAs and repetitive DNA

Small interfering RNAs (siRNAs) are related to retro-elements in plants and fungi: In plants they are known to silence retro-elements (e.g. [35]) and promoter regions by DNA and histone methylation (e.g. [36]). In *S. pombe *siRNA complementary to centromeric dh repeats [37] and other retrotransposon LTRs [38] are involved in heterochromatin silencing. Recently, numerous mammalian miRNAs with extensive homology to known repetitive elements were described [39], including rat *mir-333 *[9]. These and three further miRNA sequences (*mir-308*, *mir-421*, and *mir-430*) as well as *mir-220*, which is discussed in the following section, are excluded from the phylogenetic analysis. They are marked with the symbol ♠ in the summary table in the appendices found in the supplemental material.

The *D. melanogaster *and *D. pseudoobscura mir-308 *sequences reside in the last intron of the gene encoding the 23S ribosomal protein. Candidate sequences in insects were classified as simple repeats or low complexity regions by Repeatmasker [40]. Putative homologs in vertebrates were identified as LINES, SINES, MER2_type and simple repeats. None of those are associated with Rps23S. The mature sequences were not conserved between those candidates, the only feature they had in common were long stretches of A and T rich regions.

The eutherian specific *mir-421 *is located on the X-chromosome. The majority of candidates were identified as L2/LINEs elements, the remaining ones as SINE/Alu (Alu, B1F), and SINE/MIR (MIRb). The locus reflects the features of repeat-derived miRNAs as described in [39]. Two L2 elements in tail-to-tail orientation form the stem of the pre-miRNA, whereas the loop consists of the poly(T) tail (here poly(A) since one of the L2s is found on the minus strand) and the short intervening sequence. In contrast, the sequences of eutherian specific microRNAs that are not related to any known retrotransposon are in most cases conserved almost perfectly among different eutherian species.

The *mir-430 *family apparently is derived from a zebrafish repetitive element of unknown type.

### Tubulin genes and mir-220

The tubulin superfamily comprises 6 families [41]. Three of them, the alpha, beta and gamma tubulins, are ubiquitous for eukaryotes and used for several phylogenetic studies within this kingdom, e.g. [42]. Multiple highly conserved alpha and beta tubulin genes are found within each species. In addition, several intronless tubulin pseudogenes were found [43,44], flanked by different repeat regions [45]. These remnants of functional genes were, for instance, used as molecular clock for investigating hominide evolution [46].

*Mir-220 *was discovered in *D. rerio *[47], where it is found in the fourth exon of an mRNA (***NM199975.1***) that appears to be related to tubulin-beta genes. It can be mapped unambiguously to the minus strand of several *D. rerio *ESTs.

The human *mir-220 *sequence was identified by homology to the experimentally verified *D. rerio *sequence. It is located in a genomic region highly conserved between several vertebrates according to the conservation track of the UCSC genome browser. On the DNA sequencing clone RP5-1189B24 (***AL030996***) this region is annotated as tubulin beta-5 (TUBB5) pseudo-gene. The *mir-220 *resides on the opposite strand of this predicted gene at a position homologous to the 5' end of exon 4 in the functional TUBB4. None of the sequences in the human ESTs of GenBank contained *hsa-mir-220*.

**Figure 5:**
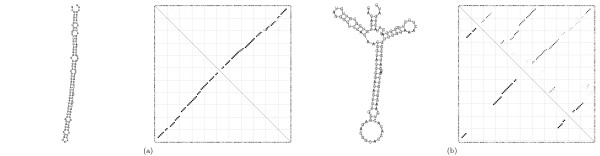
RNA secondary structures of human (a) and zebrafish (b) *mir-220 *sequences. Calculations were performed using RNAfold -p -d2 -noLP.

None of the numerous blast hits for *mir-220 *was identified as a repetitive sequence but rather appear to belong to tubulin genes and pseudogenes. Only the human sequence folds into a proper stem-loop structure, whereas the zebrafish microRNA results in a branched structure, Fig. 5. The multiple sequence alignment does not display typical features of miRNAs either. The mature sequence contains one gap in the human sequence and in addition one mismatch. Neither the loop region, nor the complementary arm, the 5' and 3' ends of the precursor are highly diverse. Furthermore, *mir-220 *would be the first microRNA to be processed from the anti-sense strand of a coding exon, a mode of transcription known so far only for cis-acting anti-sense transcripts [48].

Taking these facts together, it is conceivable that *mir-220 *is an experimental artifact. At the very least, homologous sequences in species other than zebrafish should not be interpreted as microRNAs in absence of additional evidence. We therefore disregard *mir-220 *in our further analysis.

### Distant homologies

Using blast, we have been able to identify a substantial number of microRNAs with different microRNA Registry names as homologs. As a consequence, our survey distinguishes 292 microRNA families (plus two sequences which could not be mapped to their respective genomes), while our starting point, the MR 6.0, contains 341 different family names for animal microRNAs.

In order to detect distant homologies between microRNA families that cannot be unambiguously determined from the precursor sequences, we also analyzed the mature microRNAs. Comparing alignments with shuffled sequences as described in the methods section, we obtain 95 pairs, 8 triples, and 3 quadruples of microRNA families at a *z*-score cutoff value of 3.0. Among them is in particular the entire **mir-134 **cluster, which can also be identified based on the precursor sequences Fig. 7.

While mature microRNAs are much better conserved than the rest of the precursor sequences, they are at the same time less informative because of their short length (≈ 22nt). It is therefore not warranted to conclude that mature miRNAs which exhibit statistically significant similarities (as measured by the *z*-score of their alignment) are true homologs. The observed similarities could also have arisen through convergent evolution. For example, the first 8 nucleotides of the mature sequences show highly conserved patterns between certain families of microRNAs that regulate target genes of the *Notch *signaling pathway. These motifs have been characterized as GY-box, Brd-box, and K-box [49]. In general, the corresponding pre-miRNA sequences are too divergent to conclude that they derive from a common ancestral sequence.

**Figure 6:**
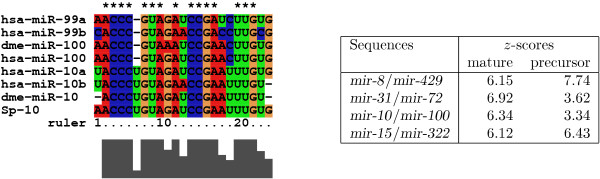
Some microRNA families, such as the *mir-10 *and *mir-100 *(left), exhibit very similar mature miRNA sequences, while their precursor sequences show little sequence similarity. Right: A table of alignment *z*-score for both mature and precursor sequences summarizes the four most likely candidates for distance homologies. While the *mir-8*/*mir-429 *pair is most likely a true homolog, the other three pairs are unconvincing, see text.

**Figure 7:**
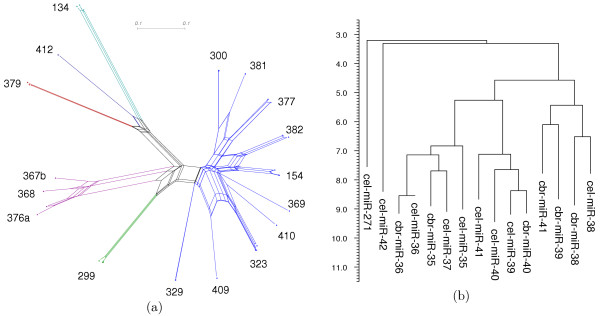
(a) All microRNAs in the **mir-134 **cluster appear to have arisen from a common ancestral sequence. The individual paralog groups have diverged rapidly in the ancestor of extant eutherian. Surprisingly, there is very little sequence variation between human and rodents in each of the paralog groups. The six families of alignable microRNAs are indicated in color. (b) WPGMA dendrogram derived from pairwise *z*-scores of the members of the **mir-35 **cluster. The analysis of the mature sequences demonstrates that the members of the cluster probably have arisen by means of tandem duplications.

In four cases we find strong evidence for homology that was not detectable directly by means of blast, see Fig. 6. The first two of these cases identify putative orthologs in distant clades:

Arthropod-specific *mir-8 *is related with vertebrate-specific *mir-429*. Their mature sequences are 74% identical, the combined stem regions still have about 60% sequence identity. A re-examination of the full precursor sequences leads us to conclude that arthropod *mir-8 *and vertebrate *mir-429 *are indeed orthologs.

Similarly, the mature sequences suggest that the nematode microRNA *mir-72 *is possibly homologous with *mir-31 *in arthropods and vertebrates. However, the full precursor sequences cannot be aligned convincingly. The *z*-score of *z *= 3.62 is only marginally significant. We hence (conservatively) count *mir-31 *and *mir-72 *as different families.

In a few more cases, distant putative paralogs can be detected using the *z*-score measure.

A particularly interesting case is the similarity between the *Hox*-cluster associated *mir-10 *and the *mir-100 *family, which is part of the **let-7 **cluster. They are annotated as members of the single microRNA precursor family RF00104 in the Rfam database. The mature sequences are 72% identical, the combined stem-regions share about 50% of the nucleotides, while the alignment of the complete precursor sequences is at the border of significance. In contrast, we cannot confirm that *mir-51 *and *mir-57 *are putative homologs of *mir-10/mir-100*. While it is likely that the *mir-10 *and *mir-100*, two old and developmentally important microRNAs, are homologous, we still treat them conservatively as distinct families in all statistics reported in this contribution. In any case, the putative duplication from which the *mir-10 *and *mir-100 *families arose, would date back at least to the eubilaterian ancestor.

The alignment *z*-scores of the *mir-15 *and *mir-322 *precursor sequences also hint a distant homology. The human ortholog of *mir-322*, designated as *hsa-mir-424 *is located 0.4 M downstream of the extra copy of the **mir-17 **cluster [6] located at the mammalian X-chromosome. It partially overlaps in its 3' end with the known mRNA BC007360, of which the third exon is annotated as Ensembl Gene ENSG00000165705 with predicted homologs in chimp (ENSPTRG00000022288) and cow (ENSBTAG00000001876). The entire region appears to be specific to mammals, as no homologs in the chicken genome can be found in the UCSC genome browser, although synthenic regions upstream and downstream of the miRNA exist on chicken chromosome 4. These genes as well as intergenic regions show roughly two to three-fold compression in chicken, but the region containing the miRNA is 18 times longer in human. The synthenic region of human Xq on chicken chromosome 4p corresponds to a microchromosome in all other birds but *Galliformes*, indicating a spot of heavy rearrangements, which might explain missing sequences [50]. The available information is insufficient to determine unambiguously whether *mir-322/mir-424 *is a true homolog of *mir-15 *that arose during the processes that lead to the assembly of the eutherian X-chromosome. Thus we conservatively count *mir-322/mir-424 *and *mir-15 *as distinct microRNA families.

## Discussion

The systematic search for orthologs and paralogs of known animal microRNAs provides a suitable basis for studying their evolution. While microRNAs exist both in multicellular animals and multicellular plants, there is no evidence that particular microRNA sequences are homologous between the kingdoms. Here we systematically study the evolution of the more than 200 known animal microRNA families. Our analysis identified a substantial number of known microRNAs as homologs despite the fact that they have different names in the MicroRNA Registry. In a few additional cases, there is at least circumstantial evidence for distant homologies. Nevertheless, vertebrate genomes contain almost 200 distinct microRNA families that do not share significant sequence homology. As most of these families cannot be traced back to an ancestral bilaterian, we have to conclude that microRNAs can arise as *de novo *genes.

The evolution of the metazoan microRNA complement is therefore characterized by four processes:

(1) *De novo *appearance of novel miRNAs. Some of these sequences arise as additional members of existing clusters. In [6], a model is proposed for this expansion process based on the fact that hairpins are very abundant RNA secondary structures. Such innovations occur throughout animal innovation. They are concentrated in the bilaterian ancestor, the vertebrate ancestor, and the eutherian ancestor. The data are at present insufficient to determine whether such periods of increased microRNA innovation also happened in invertebrate lineages. However, a small number of microRNAs are derived from repetitive elements.

(2) Tandem duplications are a frequent mechanism accounting in particular for the expansion of microRNA clusters. Such local duplications are also strongly overrepresented in the vertebrate ancestor, and at the origin of placental mammals. In the latter case, most duplications are associated with the **mir-134 **cluster.

(3) Non-local duplications of microRNAs are almost exclusively associated with the genome-wide duplication(s) in the vertebrate [51] and the teleost ancestor [52], respectively.

(4) A small class of non-local duplications is not associated with genome-wide duplication events. The only invertebrate example is the duplication of *mir-9 *in arthropods. In the ancestral eutherian we find 6 such events, mostly associated with the formation of the X-chromosome. Indeed, the mammalian X chromosome has generated and recruited a disproportionately high number of functional retroposed genes [53], which might also have affected some microRNA genes, including the X-chromosomal copy of the **mir-17 **cluster.

## Conclusion

The expansion of the microRNA repertoire is consistent with the idea that the complex metazoan genomes require an additional level of regulators [54,55]. As one would expect from such a model, dramatic expansions of the microRNA repertoire appear to be associated with major bauplan innovations: in ancestral bilaterians, ancestral vertebrates, and with the advent of (placental) mammals.

## Methods

### Sequence searches

The protocol essentially follows [6], see [7] for a detailed description with examples. For RNA folding we used the programs contained in the Vienna RNA Package [56,57]. Sequence searches were performed locally using NCBI blast (version 2.2.6) [58] with default settings and an *E*-value cutoff of *E *< 0.01, alignments were computed with clustalw [59] and visualized using clustalx [60]. The non-stringent *E*-value cutoff was chosen in order to minimize false negatives, false positives at this stage do not pose a problem because of the stringent filters in the subsequent stages of the analysis.

All metazoan microRNA precursor sequences contained in the MR 6.0 (May 2005) were blasted against the available metazoan genomes (see list in the appendices, supplemental material) as well as a few protist genomes. The resulting blast hits were extracted from the database such that the retrieved sequences had approximately the same length as the query sequences. Multiple alignments of known microRNA sequences and putative homologs were constructed using clustalw and visually inspected for unrelated sequences or sequences not sharing a well conserved mature miRNA. The aligned sequences were trimmed to closely match the length of the known homologs from the MicroRNA Registry and then realigned.

RNAalifold [61] was used to verify the hairpin structure of the consensus fold. In some cases, sequences that deviated from the phylogenetic expectation were folded separately and tested for thermodynamic stability using the randfold program [62]. In cases where candidate sequences had to be removed, the alignments were recomputed.

MicroRNAs for which only nematode sequences were known, were blasted against all vertebrate and all arthropod genomes with a cutoff of only *E *≤ 0.1. Cases in which the blast hits consistently overlap with the mature microRNA were considered further. Next we considered the vicinity of the blast hit and checked whether it is conserved in vertebrates or arthropods, respectively. This leaves only *mir-86 *(vertebrates) and *mir-72 *(arthropods) as possible candidates with unknown orthologs. In both cases the candidate sequences do not form a conserved hairpin structure so that we conclude that they are probably not homologous microRNAs.

The blast searches were complemented by searches for distant homologs similar to the procedure described in [63].

The consensus secondary structure of the final alignments of the known microRNAs and their homologs as determined above was computed using RNAalifold and converted into a search pattern for the erpin program [64]. For each microRNA, we determined the subtree spanned by known sequences and blast hits. Using erpin, we then screened within this subtree those genomes in which we did not find a blast hit, as well as all genomes from sister groups under plausible phylogenetic assumptions. In particular, both insects and nematodes were investigated for microRNAs that could be found in all vertebrates. Conversely, for apparently insect- or nematode-specific sequences we checked the other invertebrate clade as well as a sample of vertebrate genomes.

erpin searches were repeated with different score thresholds in order to balance sensitivity versus specificity, such that for each query model no more than a few dozen candidates per genome were returned. These candidates were filtered in the following way: (1) RNAfold was used to compute the secondary structure. Sequences were removed from the candidate list if removal of at most 4 base pairs did not result in an unbranched stem-loop structure. (2) Sequences passing the first test were removed if their *p*-value for structural stabilization computed by randfold-2 [62] exceeded 0.03. (3) The remaining sequences were aligned with the original search profiles. Only candidates with a significant sequence similarity according to visual inspection were retained. (4) We finally used the erpin candidates in blast searches against the remaining genomes. Candidates without a plausible phylogenetic conservation were rejected.

### Phylogenetic analysis

We pragmatically define a microRNA family as a collection of microRNA precursors for which we can construct a plausible sequence alignment using a global alignment tool such as clustalw, i.e., for which sequence homology is unambiguous. Gene phylogenies were reconstructed using the neighbor-net method [65] as implemented in SplitsTree4 [66]. The approximate trees were checked for consistency with accepted phylogenetic hypotheses.

For all microRNA precursors for which paralogs are known or have been detected in our survey, we attempted to reconstruct the duplication history from the gene tree. In the case of physically linked microRNA clusters we additionally verified that the gene phylogenies of the individual cluster members were consistent with the linkage information. We checked in particular for evidence of additional, relatively recent duplication events of microRNAs in teleosts relative to the tetrapods.

### Detection of distant homologies

In order to identify distant sequence similarities between precursor miRNAs from different paralog groups we computed a similarity score based on the significance of the alignment score: The identity score *s*(*I*, *J*) for the pairwise alignment of two precursor miRNAs *I *and *J *was computed using the implementation of the fast approximate Wilbur-Lipman algorithm [67] from the clustalw program. Then the mean identity score *m *and the variance *ν *of randomly permuted sequences were estimated by sampling. The *z*-score *z*(*I*, *J*) = (*s*(*I*, *J*) - *m*)/v
 MathType@MTEF@5@5@+=feaafiart1ev1aaatCvAUfKttLearuWrP9MDH5MBPbIqV92AaeXatLxBI9gBaebbnrfifHhDYfgasaacH8akY=wiFfYdH8Gipec8Eeeu0xXdbba9frFj0=OqFfea0dXdd9vqai=hGuQ8kuc9pgc9s8qqaq=dirpe0xb9q8qiLsFr0=vr0=vr0dc8meaabaqaciaacaGaaeqabaqabeGadaaakeaadaGcaaqaaiabdAha2bWcbeaaaaa@2E3C@ was used as a convenient measure of similarity between the sequences *I *and *J*.

We used the very well-conserved mature microRNAs to identify possible homologies that had not been reported previously. In the first step, clustalw alignments were used to determine groups of mature microRNAs with pairwise identities in excess of 70%. From the resulting 291 groups, which approximately correspond to the microRNA families, we determined consensus sequences. For these we computed all pairwise alignment *z*-scores using 100 shuffled sequences. Subclusters with pairwise *z*-scores better than *z *= 3.0 were extracted. In order to check the stability of the procedure, *z*-score matrices for these subclusters were re-calculated from 1000 shuffled sequences. This method produces robust similarity scores in regimes where reliable global alignments cannot be obtained [6]. Standard WPGMA clustering [68] was then used to estimate a dendrogram from the *z*-scores.

## Authors' contributions

This work is based on the results of two bioinformatics computer lab courses held at the Universities of Vienna and Leipzig in the Winter Semester 2004/2005. The following students contributed their preliminary analysis of 10–20 microRNA families to this work:

Sten Heinze, Alexander "muppet" Donath, Sven Findeiβ, Stephanie Keller, Kevin Peter, Julian Jöris, Jakob Mühmel, Marco Dienelt, Lisa Hellwig, Maiko Lohet, Holger Schmidtchen, Nick Jagiella, Andrej Aderhold, Paul-Robert Kästerer, Thomas Skodawessely (in Leipzig), Martina Hödl, Bernhard Wurzinger, Camille Stephan-Otto Attolini, Ulrich Omasits, Sebastian Krüttner, Regina Anzengruber, Daniela Lenek, Gregor Neumayr, Sebastian Schmittner, Reinhard Wohlfart (in Vienna). The computer lab work was supervised by C.F., J.H., M.L., K.M., and A.T. Ch.F., I.L.H., and P.F.S. planned the courses and supervised the supervisors. A.T. contributed a re-analysis of the **mir-17 **cluster. J.H., M.L., K.M., C.F., and P.F.S. collected and cross-checked the student contributions. J.H., M.L., K.M., and P.F.S. computed the summary statistics, J.H. and A.T. investigated the distant homologies, A.T. analyzed the repeat associated microRNAs, and K.M. organized the supplemental material. All authors collaborated closely in preparing this manuscript.

## Supplementary Material

Additional file 1 Appendix A: MicroRNA distribution across metazoa Click here for file

Additional file 1 Appendix B: Distribution of insect-specific microRNAs
Click here for file

## Figures and Tables

**Figure 1:**
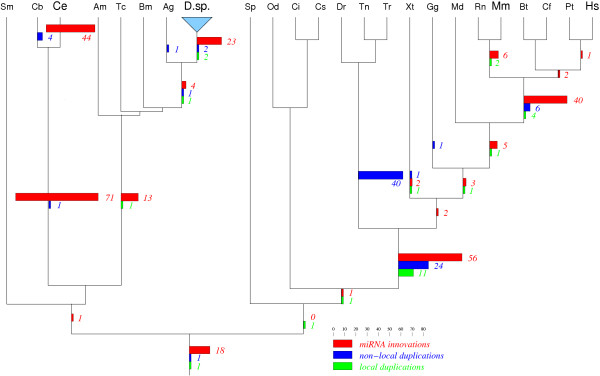
Innovations of microRNAs, tandem duplications, and non-local duplications of microRNA genes are unevenly distributed in metazoan phylogeny. Indeed, non-local duplications occur almost exclusively in the ancestral vertebrate and teleosts, resp., in accordance with the 2R/3R model. Species for which large experimental screens for microRNAs have been performed are indicated by a larger font. The phylogenetic tree is based on a recent multi-gene analysis of the major bilaterian groups [69], and the phylogeny of holometabolous insects [70].

**Figure 2:**
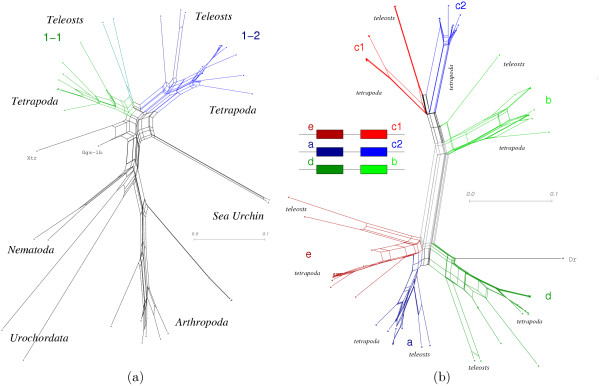
(a) Phylogenetic network of *mir-1 *sequences. Despite the short sequences, the major clades are well separated in this phylogenetic network: there are two vertebrate groups, *mir-1-1 *and *mir-I-2*, both of which show a tetrapod and a teleost branch; arthropoda and nematoda are also clearly separated; only the basal deuterostomes do not fit very well due to their diverged sequences. (b) Phylogenetic network of **mir-30 **sequences, which occur in three clusters each consisting of two miRNAs genes (see inset). A tandem duplication of the ancestral *mir-30 *sequence gave rise to a single cluster which was duplicated subsequently. Not all details of the duplication history can be resolved due to the short sequence length. It is clear, however, that the duplication events pre-dated the last common ancestor of tetrapoda and teleosts. It is plausible to associate these cluster duplications with the genome duplications at the origin of the vertebrate lineage. Networks were reconstructed using the neighbor net method.

**Table 1:** Summary statistics of the dataset used in this study. MicroRNA genes detected by homology search relative to the contents of the MR 6.0.

Genome	MR 6.0	known	new	all
hsa	227	215+12	23	238
ptr	-	0	183	183
cfa	6	6	195	201
bta	-	0	138	138
mmu	230	215+17	26	241
rno	191	180+6	39	219

mdo	-	0	139	139
gga	122	122	17	139
xla/xtr	(7)	(7)	126	133

tru	-	0	171	171
tni	-	0	179	179
ola	-	0	152	152
dre	33	60	205	265

spu	-	0	40	40
cin	-	0	6	6
csa	-	0	3	3
odi	-	0	5	5

dme	78	78	0	78
dps	73	72	0	72
dya	-	0	74	74
dan	-	0	64	64
dvi	-	0	67	67
dmo	-	0	69	69
aga	38	42	10	52
tca	-	0	24	24
ame	25	26	12	38
bmo	-	0	17	17

cel	116	117	2	119
cbr	79	82	3	85

sma	-	0	4	4

Σ		1222	1993	3215

The set of "known" microRNAs differs in some cases from MR 6.0 because some database entries could not be mapped to the current genome assembly, or mapped to more than one genomic locus. The **mir-134 **cluster is excluded from this list (its known members are indicated separately for human, mouse and rat in the MR 6.0 column). The last column ("all") provides the statistics for the data set provided in the electronic supplement, the column "new" lists all those pre-miRNA sequences that were detected by homology search and are contained in MR 6.0. For Xenopus 7 microRNAs were reported for *Xenopus laevis*, a close relative of the sequenced *Xenopus tropicalis*.

**Species abbreviations**.

Mammals: hsa, Hs: *Homo sapiens*; ptr, Pt: *Pan troglodytes*; cfa, Cf: *Canis familiaris*; bta, Bt: *Bos taurus*; mmu, Mm: *Mus musculus*; rno, Rn: *Rattus norvegicus*; mdo, Md: *Monodelphis domesticus*; other tetrapods: gga, Gg: *Gallus gallus*; xla: *Xenopus laevis*; xtr, Xt: *Xenopus tropicalis*; teleost fishes: tru, Tr: *Takifugu rubripes*; tni, Tn: *Tetraodon nigroviridis*; dre, Dr: *Danio rerio*; basal deuterostomes: spu, Sp: *Strongylocentrotus purpuratus*; cin, Ci: *Ciona intestinalis*; csa, Cs: *Ciona savignyii*; odi, Od: *Oikopleura dioica*; insects: dme, Dm: *Drosophila melanogaster*, dps, Dp: *Drosophila pseudoobscura*, dya, Dy: *Drosophila yakuba*, dan, Da: *Drosophila ananassae*, dvi, Dv: *Drosophila viridis*, dmo, Do: *Drosophila mohavensis*, aga, Ag: *Anopheles gambiae*, tca, Tc: *Tribolium castaneum*, ame, Am: *Apis mellifera*, bmo, Bm: *Bombyx mori*, nematods: cel, Ce: *Caenorhabditis elegans*, cbr, Cb: *Caenorhabditis briggsae*, platyhelmint: sma, Sm: *Schistosoma mansoni*.

**Table 2:** Vertebrate microRNA clusters. The table lists the maximal number of microRNAs in a single copy of the cluster ("Members"), the maximal number of non-homologous microRNAs in a single copy ("Families"), and the maximal number of paralogous cluster copies in any of the investigated genomes.

Cluster	Members	Families	Paralogs
**let-7**	3	3	18
**mir-1**	2	2	4
**mir-2**	4	2	5
**mir-3**	9	6	3
**mir-9**	4	3	7
**mir-12**	2	2	1
**mir-15**	2	1	5
**mir-17**	6	3	9
**mir-23**	3	3	6
**mir-29**	3	2	8
**mir-30**	2	1	3
**mir-34**	2	2	3
**mir-35**	7	7	1
**mir-42**	3	3	2
**mir-46**	2	2	5
**mir-51**	4	4	1
**mir-54**	3	3	1
**mir-61**	2	2	1
**mir-64**	4	4	1
**mir-73**	2	2	1
**mir-77**	2	1	1
**mir-96**	3	3	2
**mir-105**	3	1	1
**mir-127**	2	1	* 2
**mir-130**	2	2	5
**mir-132**	2	1	2
**mir-134**	>50	6	* 1
**mir-141**	2	1	* 2
**mir-143**	2	2	1
**mir-181**	2	1	8
**mir-191**	2	2	* 1
**mir-192**	2	2	2
**mir-202**	2	1	1
**mir-204**	2	1	3
**mir-216**	2	1	2
**mir-221**	2	1	4
**mir-232**	2	1	1
**mir-249**	2	1	1
**mir-275**	2	2	1
**mir-276**	2	1	1
**mir-290**	6	1	6
**mir-296**	2	1	2
**mir-302**	5	2	5
**mir-310**	4	4	1
**mir-344**	3	1	1
**mir-357**	2	2	2
**mir-374**	3	2	1
**mir-450**	3	1	1

* part of the human **mir-134 **cluster experimentally investigated in [33]. In the same study it is reported that *mir-144 *and *mir-224 *are also parts of clusters with additional microRNAs that do not have orthologs in the MR 6.0.
