# Supplementary material for: The expansion of the metazoan microRNA repertoire
Source: BMC Genomics. 2006 Feb 15;7:25. doi: 10.1186/1471-2164-7-25 (PMC1388199; doi:10.1186/1471-2164-7-25)
Supplement: Additional file 1 — Appendix A: MicroRNA distribution across metazoa [file 1471-2164-7-25-S1.pdf]

Appendix A: MicroRNA distribution across metazoa

miR	Pr	Ro	Eu	Md	Gg	Xt	Tf	b.d.	Ar	Ne	Sm	PF	Remark
bantam									•				
iab-4									•				[9]
let-7	■	■	■	■	■	■	⧫*		•	•			+98 [5]
100	■	■	■	■	■	■	⧫*		•				+99
125	■	■	■	■	■	■	⧫*	•	•				
lin-4										•			
lsy-6										•			
1	■	■	■	■	■	■	■*	•	•	•	○		+206
133	■	■	■	■	■	■	⧫*	•	•	○			
2									■	•			
13									•				
71										•			
3									■				+309
4									•				
5									•				
6									■				
286									•				
7	■	■	■	■	■	■	⧫*	•	•				
8	•	•	•	•	•	•	•		•		•′		+429
9	■	■	■	■	■	■	⧫*	•	■	•	○		+79
306									•				
10	■	■	■	■	■	■	⧫*	•	•				[9]
11									•				
12									•				
304									•				
13	→ **mir-2** cluster												
14									•				
15	■	■	■	■	■	■	⧫*						+16,195
16	*mir-15* paralog												
17	■	■	■	■	■	■	⧫*						+18,20,93,106 [6]
18	*mir-17* paralog												
19	■	■	■	■	■	■	⧫*						
20	*mir-17* paralog												
92	■	■	■	■	■	■	⧫*	•	■	•			+25,235
21	•	•	•	•	•	-	⧫*						
22	•	•	•	•	•	•	⧫*						
23	■	■	■	■	•	■	⧫*						
24	■	■	■	■	•	•	⧫*						
27	■	■	■	■	■	•	⧫*						
25	*mir-92* paralog → **mir-17** cluster												
26	■	■	■	■	•	•	⧫*						
27	→ **mir-23** cluster												
♠28	■	■	■	■									+151 LINE L2

. . . continued on next page . . .

. . . continued from previous page

miR	Pr	Ro	Eu	Md	Gg	Xt	Tf	b.d.	Ar	Ne	Sm	PF	Remark
29	■	■	■	■	■	■	◆*		●				+285
30	■	■	■	■	■	■	◆*						
31	●	●	●	●	●	●	●*	●	■				
32	●	●	●	●	●								
33	●	●	●	●	●	●	◆	●	●				
34	■	■	■	■	■	●	●*		●	●			
277									●				
35										●			
36										●			
37										◎			
38										●			
39										●			
40										●			
41										●			
42										●			
43										●			
44										●			
45										■			
46										●			
281								■	■	●′			
47										●			
48										●			
241										●			
49										●			
50										●			
51										●			
52										●			
53										◎			
232										■			
54										◎			
55										●			
56										◎			
57										●			
58										●			
270										◎			
59										◎			
60										●			
61										●			
250										●			
62										●			
63										◎			
64										●			
65										◎			
66										◎			
229										◎			

. . . continued on next page . . .

. . . *continued from previous page*

miR	Pr	Ro	Eu	Md	Gg	Xt	Tf	b.d.	Ar	Ne	Sm	PF	Remark
67										●			
68										◎			
69										◎			
70										●			
71	→ **mir-2** cluster												
72										●			
73										●			
74										●			
75										●			
76										◎			
77										■			
78										◎			
79	*mir-9* paralog → **mir-9** cluster												
80										●			
238										◎			
81										●			
82										●			
83										●			
84										●			
85										●			
86										●			
87									●	●			
90										●			
92	→ **mir-17** cluster												
93	*mir-17* paralog → **mir-17** cluster												
♠95	■	■	●	●									LINE L2
96	●	●	●	●	-	●	♦*						
182	●	●	●	●	-	●	♦*						
183	●	●	●	●	●	●	♦*	●					
98	*let-7* paralog												
99	*mir-100* paralog → **let-7** cluster												
100	→ **let-7** cluster												
101	■	■	■	■	●	■	■*						
103	■	■	■	■	■	■	■*						=107(rc)
105	■	●	■										
106	*mir-17* paralog → **mir-17** cluster												
107	r.c. of *mir-103*												
108	r.c. of *mir-365*												
122	●	●	●	●	●	●	●*						
124	■	■	■	■	■	■	♦*	●/■	●	●	●		
125	→ **let-7** cluster												
126	●	●	●	●	●	●	●*						
127	●	●	●										
136	●	●	●										
128	■	■	■	■	■	■	■*						

. . . *continued on next page* . . .

. . . continued from previous page

miR	Pr	Ro	Eu	Md	Gg	Xt	Tf	b.d.	Ar	Ne	Sm	PF	Remark
129	■	■	■	■	-	■	♦*						
130	■	■	■	■	■	■	♦*						+301
132	■	■	■	■	-	■	♦*						+212
133	→ **mir-1** cluster												
134	●	●	●										
154	■	■	■										+300, 323, 329, 369, 377, 381, 382, 409, 410
368	■	■	■										+376
299	●	●	●										
379	■	■	■										+380,411
412	●	●	●										
135	■	■	■	■	■	■	■*						
136	→ **127** cluster												
137	●	●	●	●	●	■	♦*						
138	■	■	■	●	■	■	■*						
139	●	●	●	●	●	●	●*						
140	●	●	●	●	●	●	●*						
141	■	■	■	■	■	■	■*						+200
142	●	●	●	●	●	●	♦*						
143	●	●	●	●	●	●	●*						
145	●	●	●	●	-	●	●*						
144	●	●	●	●	●	●	●*						
145	→ **mir-143** cluster												
146	●	●	●	●	●	●	*						
147	●		●										human, dog
148	■	■	■	■	●	■	■*						+152
149	●	●	●										
150	●	●	●				*						
151	*mir-28* paralog												
152	*mir-148* paralog												
153	■	■	■	■	●	■	♦*						
154	→ **mir-134** cluster												
155	●	●	●	●	●	●	●*						
181	■	■	■	■	■	■	♦*						+213
182	→ **mir-96** cluster												
183	→ **mir-96** cluster												
184	●	●	●	●	●	-	♦*	●	●				
185	●	●	●										
186	●	●	●	●									
187	●	●	●	●	●	●	●*						
188	●	●	●										
190	●	●	●	●	●	-	♦*						
191	●	●	●	●									
425	●	●	●	●									
192	■	■	■	●	●	-	●*						+215

. . . continued on next page . . .

... continued from previous page

miR	Pr	Ro	Eu	Md	Gg	Xt	Tf	b.d.	Ar	Ne	Sm	PF	Remark
194	■	■	■	●	●	●	●*						
193	●	●	●	●	●	-	♦*						
194	→ **mir-192** cluster												
195	*mir-15* paralog												
196	■	■	■	■	■	■	♦*						[9]
197	●		●										
198	●												
199	■	■	■	■	■	●	■*						
200	*mir-141* paralog												
201		●											
202	●	●	●	●	●	■	●						
203	●	●	●	●	●	●	●*						
204	■	■	■	■	■	■	■*						+211
205	●	●	●	●	■	●	●*						
206	*mir-1* paralog												
207	●	●	●										
208	●	●	●	●									
210	●	●	●	●	-	●	●*		●				
211	*mir-204* paralog												
212	*mir-132* paralog												
213	*mir-181* paralog												
214	●	●	●	●	-	●	♦*						
215	*mir-192* paralog												
216	●	●	●	●	●	●	●*						
217	●	●	●	●	●	●	●*						
218	■	■	■	■	■	■	●*						
219	■	■	■	■	●	●	♦*		●				
♠220													tubulins
221	■	■	■	■	■	■	♦*						+222
222	*mir-221* paralog												
223	●	●	●	●	●	●	●*						
224	●	●	●										
228										●			
229	→ **mir-64** cluster												
230										●			
231										●			
232	→ **mir-51** cluster												
233										●			
234										●			
235	*mir-92* paralog [6]												
236										●			
237										◎			
238	→ **mir-80** cluster												
239										■			
240										●			

... continued on next page ...

. . . continued from previous page

miR	Pr	Ro	Eu	Md	Gg	Xt	Tf	b.d.	Ar	Ne	Sm	PF	Remark
241	→ **mir-48** cluster												
242										◎			
243										◎			
244										•			
245										•			
246										•			
247										◎			
248										•			
249										•			
359										◎			
250	→ **mir-61** cluster												
251										•			
252										•			
253										•			
254										•			
255										•			
256										◎			
257										◎			
258										◎			
259										•			
260										◎			
261										◎			
262										◎			
263									■				
264										◎			
265										◎			
266										◎			
267										◎			
268										•			
269										◎			
270	→ **mir-58** cluster												
271										◎			
272										◎			
273										◎			
274									•				
275									•				
305									•				
276									■				
277	→ **mir-34** cluster												
278									•				
279									•				
280									•				
281	→ **mir-46** cluster												
282									•				
283									•				

. . . continued on next page . . .

… *continued from previous page*													
miR	Pr	Ro	Eu	Md	Gg	Xt	Tf	b.d.	Ar	Ne	Sm	PF	Remark
284									•				
285	*mir-29* paralog												
286	→ **mir-3** cluster												
287									•				
288									•				
289									•				
290	■	■	■										+291-295,371-373[34]
291	*mir-290* paralog												
292	*mir-290* paralog												
293	*mir-290* paralog												
294	*mir-290* paralog												
295	*mir-290* paralog												
296	•	•	•										
298		•											
♠297		■											low compl.
298	→ **mir-296** cluster												
299	*mir-154* paralog→ **mir-134** cluster												
300	*mir-154* paralog→ **mir-134** cluster												
301	*mir-130* paralog												
302	■	■	■	■	•								
367	•	•	•	•	•								
303									•				
304	→ **mir-12** cluster												
305	→ **mir-275** cluster												
306	→ **mir-9** cluster												
307									•				
308									•				
309	*mir-3* paralog → **mir-3** cluster												
310									•				
311									•				
312									•				
313									•				
314									•				
315									•				
316									•				
317									•				
318									•				
320	■	•	•										
322	•	•	•										
323	*mir-154* paralog→ **mir-134** cluster												
324	•	•	•										
♠325	•	•	•										LINE L2
326	•	•	•										
♠327		■											LINE L2
328	•	•	•										
													… *continued on next page* …

. . . continued from previous page

miR	Pr	Ro	Eu	Md	Gg	Xt	Tf	b.d.	Ar	Ne	Sm	PF	Remark
329	*mir-154* paralog→ **mir-134** cluster												
330	•	•	•										
331	•	•	•										
♠333		•											B2-related [9]
335	•	•	•										
336		•											
337	•	•											
338	•	•	•	•	•	-	♦*						
339	•	•	•										
♠340	•	•	•										
♠341		•											
342	•	•	•										
343		•											
344		■											
345	•	•	•										
346	•	•	•										
347		?											rat only
349		•											
350	(■)	•	•										insertion
351		•											
352		?											rno-mir-352
353										•			
354										•			
355										•			
356										•			
357										■			
358										•			
359	→ **mir-249** cluster												
360										•			
361	•	•	•										
365	■	■	■	•	•	•	•*						=108rc
367	→ **mir-302** cluster												
368	→ **mir-134** cluster												
369	*mir-154* paralog → **mir-134** cluster												
370	•	•	•										
371	*mir-290* paralog → **mir-290** cluster												
372	*mir-290* paralog → **mir-290** cluster												
373	*mir-290* paralog → **mir-290** cluster												
374	■	■	■										
421	•	•	•										
375	•	•	•	•	•	-	♦*						
376	*mir-168* paralog → **mir-134** cluster												
377	*mir-154* paralog → **mir-134** cluster												
378	•	•	•										
379	→ **mir-134** cluster												

. . . continued on next page . . .

. . . continued from previous page													
miR	Pr	Ro	Eu	Md	Gg	Xt	Tf	b.d.	Ar	Ne	Sm	PF	Remark
380	*mir-379* paralog → **mir-154** cluster												
381	*mir-154* paralog → **mir-134** cluster												
382	*mir-154* paralog → **mir-134** cluster												
383	•	•	•	•	•	•							
384	•	•	•										
392										◎			
409	*mir-154* paralog → **mir-134** cluster												
410	*mir-154* paralog → **mir-134** cluster												
411	*mir-379* paralog → **mir-134** cluster												
412	→ **mir-134** cluster												
421	→ **mir-374** cluster												
422	•		•										
423	•	•	•										
424	= *mir-322*												
425	→ **mir-191** cluster												
427						•							frog only
428						•							frog only
429	*mir-8* paralog												
♠430							■						zebrafish only
448	•	•	•										
449	•	•	•	•									
450	■	■	■										

Pr: primates, Ro: rodents, Eu: other eutherian mammals (Cf, Bt), Md: oppossum, Gg: chicken, Xt: frog Tf: teleost fishes p.d.: basal deuterostomes (Ci, Cs, Od, or Sp) Ar: Arthropoda (Drosophilids, Anopheles, honeybee) Ne: Nematoda Sm: Schistosoma mansoni PF: protists, fungi, etc.

Symbolds: • single copy microRNA, ■ multiple paralogous, ○ homologs found using `erpin` but not by `blast` search, •′ homologs found only with a non-restrictive blast search $E < 0.1$ and comparison of the match position with the mature microRNA. ◎ single microRNA in *C. elegans* without homolog in *C. briggsae*

♠ associated with a repetitive element according to [39]

♦ evidence for additional duplications in teleosts

* zebrafish homolog reported in [18] and or [21].

? reported in `MicroRNA Registry 6.0` but does not map to the current genome assemblies.
