# Supplementary material for: The expansion of the metazoan microRNA repertoire
Source: BMC Genomics. 2006 Feb 15;7:25. doi: 10.1186/1471-2164-7-25 (PMC1388199; doi:10.1186/1471-2164-7-25)
Supplement: Additional file 1 — Appendix B: Distribution of insect-specific microRNAs [file 1471-2164-7-25-S2.pdf]

Appendix B: Distribution of insect-specific microRNAs

miR	Dme	Dya	Dan	Dps	Dmo	Dvi	Aga	Ame	Bmo	Tca
bantam	●	●	●	●	●	●	■	●	●	●
iab-4	●	●	●	●	●	●	●	●	●	●
3	■	■	■	■	●	●				
4	●	●	●	●	●	●				
5	●	●	●	●	●	●				
6	■	■	■	■	■	■				
11	●	■	●	●	●	●	●			
12	●	●	●	●	●	●	●	●		●
13	■	■	■	■	■	■	■	●		●
14	●	●	●	●	●	●	●	●	●	●
263	■	■	■	■	●	■	■	●	●	●
274	●	●		●	●	●				
275	●	●	●	●	●	●	●	●	●	●
276	■	■	■	■	■	■	■	●	●	●
277	●	●	●	●	●	●	●	●		●
278	●	●	●	●	●	●	●	●		
279	●	●	●	●	●	●	●	●		●
280	●	●	●	●	●	●				
282	●	●	●	●	●	●	●	●		●
283	●	●	●	●	●	●	●	●	●	
284	●	●	●	●	●	●				
286	●	●	●	●	●	●	●			
287	●	●	●	●	●	●				
288	●	●	●	●	●	●				
289	●	●	●	●	●	●				
303	●									
304	●	●	●	●	●	●				
305	●	●	●	●	●	●	●	●	●	●
306	●	●	●	●	●	●				
307	●	●	●	●	●	●	●	●	●	●
308	●	●	●	●	●	●	●			
309	●	●	●	●		●				
310	●	●								
311	●	●								
312	●	●								
313	●									
314	●	●	●	●	●	●				
315	●	●	●	●	●	●	●	●		●
316	●	●	●	●	●	●				
317	●	●	●	●	●	●	●	●	●	●
318	●	●	●	●	●	●				

Dan: *Drosophila ananassae*, Dme: *Drosophila melanogaster*, Dmo: *Drosophila mojavensis*, Dps: *Drosophila pseudoobscura*, Dvi: *Drosophila virilis*, Dya: *Drosophila yakuba*, Aga: *Anopheles gambiae*, Ame: *Apis mellifera*, Bmo: *Bombyx mori*, Tca: *Tribolium castaneum.* For phylogenetic relationships among insects and within Drosophilids see [70] and [71], resp.
